# Perception of fattening foods in Italian children and adolescents

**DOI:** 10.1186/2193-1801-3-402

**Published:** 2014-08-04

**Authors:** Stefania Toselli, Patricia Brasili, Rocco Di Michele, Federico Spiga

**Affiliations:** Department of Biomedical and Neuromotor Sciences, University of Bologna, Bologna, Italy

**Keywords:** Body-mass index, Italy, Nutritional habits, Children, Adolescents, Obesity, Overweight, Fattening food

## Abstract

The present study aimed to analyze, in Italian children and adolescents, the beliefs about which foods are fattening, the appreciation of fattening foods, and the perception of some socio-cultural attributes of them. 244 children (F: 125, M: 119, aged 8–11) and 305 adolescents (F: 153; M: 152, aged 11–14) from Bologna, Northern Italy, were asked to indicate five fattening foods. For each of the indicated foods, a yes-no answer was required to the question: “is this food special for you?”, and to six questions concerning socio-cultural attributes of the food, which were modelled using a latent variable with two classes named “traditional” and “modern”. Pearson’s chi square tests revealed, both in boys and girls, significant associations between the age class and the foods indicated as fattening: lean meat, condiments, non-sweet fruit and vegetables were more often indicated as fattening by children than by adolescents. Overall, boys showed higher appreciation and perception of fashionability of fattening foods. Girls appreciated less bread and pasta, and indicated more often than boys these foods as fattening. The different food perception of between age classes and sexes can be respectively explained by a better dietary awareness of adolescents, and by girls worrying about their look more than boys.

## Background

Childhood overweight and obesity are relevant public health problems, rapidly growing in industrialized countries during the last decades. In Italy, obesity has emerged since the 1990s (International Obesity Taskforce 2012) and continues to increase dramatically especially among children and adolescents. The prevalence of childhood overweight and obesity is the highest in the South of Italy (overweight: 27.1%; obesity: 13.5%) and the lowest in the North (overweight: 22.9%; obesity: 5.9%) (Inran 2009). Moreover, these percentages vary according to the regions, with overweight ranging from 14.9% to 40.6%, and obesity ranging from 2.4% to 19.5% (Toselli et al. 2014, Toselli et al. 2012). The prevalence of obesity tends to decrease in the adolescence, especially among girls, as the care of body image becomes usually important at this age. Toselli et al. (2010), in a sample of adolescents from Bologna (northern Italy), reported obesity rates of 5.5% (at age 12) and 3.9% (at age 13) for boys, and of 3.2% (at age 12) and 1.1% (at age 13) for girls. A lower percentage of obesity amongst adolescent girls when compared with age-matched boys was also observed in other European countries (World Health Organization 2009), and in the central-northern Italian population (Cacciari et al. 2006).

The transition from childhood to adolescence is a period in which the weight status can change due to nutritional behaviours and lifestyle habits such as practicing or not physical activity (Haerens et al. 2010). In children, nutritional patterns are mostly affected by school meals programs and by the control of parents (Demory-Luce et al. 2004), whereas adolescents usually self-select the food they like to eat, often resulting in a change of the previous nutritional habits and of food preferences. For example, Lytle et al. (2000) showed that, when students finish primary school, fruit consumption falls by 41%, while vegetables consumption falls by 25%. In another study, (Demory-Luce et al. 2004) and colleagues (Toselli et al. 2010) observed that, when compared to children, young adults decrease the consumption of meats, fruit, desserts, candy and milk, and increase the consumption of sweetened beverages, poultry, salty, snacks, seafood, cheese, beef and condiments.

Analysing how food consumption patterns develop during childhood is an important tool to understand the pathogenesis of weight disorders. Previous studies have considered food preferences (Cook and Frank 2007, Harris 2008, Cornwell and McAlister 2011), the role of branding (Halford et al. 2007), and familiarity in dietary development (Aldridge et al. 2009, Boukthir et al. 2011). According to Ulijaszek (2007), food intake is driven psychologically by innate and cognitive factors related to the food environment. The connection between the brain and gut affects the perceived qualities of foods through specific sensations such as smell, expectation, association with pleasure or disgust. Therefore, knowing that a food is unhealthy may not necessarily result in a reduced consumption of it. On the contrary, the potential fattening effect of a food may be a factor that leads to limit the consumption of that food when one’s aim is to avoid overweight and obesity. In the Italian case, identifying the popular perception of fattening foods in children and adolescents can contribute to understand the reasons for the between-sex differences of overweight and obesity observed in these age ranges. To this aim, this study analyses which foods are believed to be fattening, the extent to which these food are appreciated, and how some socio-cultural attributes of them are perceived.

## Results and discussion

Table 1 shows the weight status/age class contingency tables in boys and girls. Overweight was more prevalent among boys (about 22% for both children and adolescents), than among girls (about 15% and 12% in children and adolescents, respectively). In both sexes, there were no significantly different weight status distributions between children and adolescents (p > 0.05). Due to the limited number of underweight and obese participants, these categories were respectively included in the normal weight and overweight categories for the subsequent analyses.

**Table 1:** **Weight status**/**age class contingency tables**

Boys		
	Children	Adolescents
Underweight	0	2
Normalweight	89	113
Overweight	26	33
Obese	4	4
**Girls**		
	**Children**	**Adolescents**
Underweight	3	4
Normalweight	100	130
Overweight	19	18
Obese	3	1

The foods indicated as fattening showed, both in boys and girls, significantly different distributions (p < 0.05) between children and adolescents (Table 2). In particular, four food categories were the most responsible for the association between the two variables: lean meat, representing 6 to 7% of all fattening foods indicated by children, and 3 to 4% of fattening foods indicated by adolescents; oils and condiments, indicated more often by adolescents (boys: 7.1%; girls: 10.9%) than by children (about 4% in both sexes); non-sweet fruit/cereals and vegetables, both food types very rarely indicated as fattening by adolescents (0 to 0.5%), but sometimes indicated by children (each category 1.5 to 2.5%). It is worth noting that, overall, bread and pasta are indicates as fattening much more often by girls than by boys (Table 2).

**Table 2:** **Percentage distributions of categories for foods indicated as fattening**

Boys					
	Overall	Children	Adolescents	Normalweight	Overweight
Chocolate and snacks	23.2	20.5	25.3	22.5	25.1
Biscuits and ice creams	18.5	19.2	18.0	18.4	18.8
Fat Meat	10.9	13.3	9.1	11.5	9.3
Filled cakes and sweets	8.0	6.7	8.9	8.3	6.9
Fried potatoes	7.5	6.6	8.2	8.1	5.4
Oils and condiments	5.6	3.7	7.1	5.1	7.2
Lean meet	5.1	7.1	3.6	5.4	4.2
Cold cuts	4.9	4.9	4.9	5.4	3.3
Bread and pasta	4.4	4.4	4.5	3.8	6.3
Pizza	4.1	4.9	3.4	4.0	4.2
Juices and drinks	2.4	2.4	2.4	2.3	2.7
Dairy products	1.5	0.8	2.0	1.4	1.8
Vegetables	1.3	2.4	0.5	1.3	1.5
Non-sweet fruits and cereals	1.0	1.8	0.3	0.7	1.8
Eggs	0.7	0.5	0.9	0.7	0.9
Fried fish	0.6	0.5	0.7	0.6	0.6
Fish	0.3	0.5	0.1	0.4	0.0
Sweet fruit	0.1	0.0	0.3	0.1	0.3
**Girls**					
	**Overall**	**Children**	**Adolescents**	**Normalweight**	**Overweight**
Chocolate and snacks	26.5	25.6	27.2	27.2	22.4
Biscuits and ice creams	16.3	17.0	15.7	15.7	19.5
Bread and pasta	9.2	9.8	8.8	8.2	15.1
Oils and condiments	8.0	4.5	10.8	7.9	8.3
Fried potatoes	7.6	6.9	8.1	7.9	5.4
Filled cakes and sweets	7.0	6.7	7.2	7.4	4.4
Fat Meat	5.4	4.8	5.9	5.6	4.4
Cold cuts	5.3	5.3	5.4	5.2	5.9
Lean meet	4.2	6.1	2.7	4.3	3.9
Juices and drinks	2.5	3.0	2.1	2.3	3.9
Pizza	2.5	3.5	1.7	2.4	2.9
Dairy products	1.8	1.6	2.0	1.9	1.5
Non-sweet fruits and cereals	1.0	1.4	0.7	1.1	0.5
Vegetables	0.9	1.6	0.4	1.0	0.5
Sweet fruit	0.6	1.1	0.3	0.7	0.5
Eggs	0.5	0.6	0.4	0.4	1.0
Fish	0.4	0.3	0.4	0.4	0.0
Fried fish	0.3	0.2	0.4	0.3	0.0

No association (p > 0.05) was found between weight status and food category, both in boys and girl. In other words, normal weight and overweight participants did not show differences regarding the foods believed to be fattening (Table 2).

Table 3 shows the percentage of “yes” responses to the question on fattening food appreciation and to six questions on socio-cultural attributes of foods. All the seven variables were associated to the food category in both boys and girls (all p > 0.05). In other words, for all the questions and in both sexes, the percentage of “yes” responses was different among at least some food categories.

**Table 3:** **Percentage of** “**yes**” **responses to the question** “**is this food special for you**? ” **and to questions on socio**-**cultural attributes of foods**

Boys							
	Special	New	Fashionable	Natural	Local	T city	T countryside
Biscuits and ice creams	65.3	18.7	44.6	28.7	11.2	35.1	11.2
Bread and pasta	80.0	8.3	31.7	73.3	16.7	23.3	10.0
Chocolate and snacks	64.3	22.6	49.0	31.5	11.1	38.2	4.5
Cold cuts	60.6	9.1	19.7	74.2	27.3	6.1	36.4
Dairy products	55.0	10.0	25.0	85.0	30.0	20.0	35.0
Eggs	80.0	10.0	20.0	70.0	0.0	10.0	20.0
Fat Meat	68.9	30.4	54.1	38.5	15.5	41.2	11.5
Filled cakes and sweets	72.2	14.8	50.9	31.5	21.3	35.2	6.5
Fish	75.0	0.0	50.0	75.0	25.0	50.0	25.0
Fried fish	50.0	37.5	12.5	50.0	0.0	37.5	37.5
Fried potatoes	80.2	33.7	60.4	24.8	9.9	48.5	6.9
Juices and drinks	56.3	34.4	46.9	18.8	15.6	37.5	9.4
Lean meet	58.0	4.3	26.1	84.1	11.6	15.9	18.8
Non-sweet fruits and cereals	69.2	23.1	30.8	69.2	15.4	23.1	30.8
Oils and condiments	32.9	9.2	26.3	69.7	3.9	22.4	23.7
Pizza	85.5	21.8	58.2	43.6	30.9	40.0	9.1
Sweet fruit	100.0	0.0	0.0	100.0	0.0	0.0	0.0
Vegetables	50.0	11.1	22.2	88.9	22.2	5.6	22.2
OVERALL	65.8	19.8	44.1	42.7	14.2	33.2	12.0
**Girls**							
	**Special**	**New**	**Fashionable**	**Natural**	**Local**	**T city**	**T countryside**
Biscuits and ice creams	61.1	13.7	35.8	30.5	15.0	24.8	8.0
Bread and pasta	55.5	7.8	15.6	55.5	18.8	14.1	14.8
Chocolate and snacks	64.1	26.4	47.3	28.8	12.0	30.7	4.6
Cold cuts	58.1	13.5	28.4	66.2	35.1	5.4	20.3
Dairy products	56.0	12.0	20.0	76.0	8.0	16.0	40.0
Eggs	57.1	14.3	28.6	85.7	14.3	42.9	14.3
Fat Meat	44.0	48.0	46.7	29.3	14.7	48.0	8.0
Filled cakes and sweets	67.0	24.7	37.1	32.0	16.5	30.9	9.3
Fish	20.0	0.0	0.0	80.0	40.0	20.0	40.0
Fried fish	25.0	100.0	25.0	50.0	50.0	75.0	0.0
Fried potatoes	68.6	34.3	50.5	27.6	11.4	41.9	4.8
Juices and drinks	60.0	42.9	60.0	17.1	17.1	37.1	2.9
Lean meet	67.8	11.9	30.5	76.3	22.0	8.5	13.6
Non-sweet fruits and cereals	35.7	7.1	35.7	71.4	42.9	14.3	35.7
Oils and condiments	41.4	8.1	25.2	66.7	16.2	7.2	18.0
Pizza	74.3	11.4	37.1	40.0	34.3	22.9	11.4
Sweet fruit	77.8	22.2	11.1	77.8	55.6	0.0	44.4
Vegetables	69.2	23.1	15.4	100.0	7.7	15.4	0.0
OVERALL	59.9	21.1	37.1	41.5	16.9	25.2	10.4

T city: Typical of the city; T countryside: Typical of the countryside.

The appreciation of fattening foods was, overall, higher in boys (65.8% of “yes” responses to the question “is this food special for you?”) than in girls (59.9%). Among the foods indicated as fattening in 1% of total cases or more, the most appreciated foods were pizza (percentage of “yes” answers to the question “is this food special for you?”: 85.5% for boys and 74.3% for girls) and fried potatoes (80.2% for boys and 68.6% for girls), whereas oils and condiments was the less appreciated foods (32.9% for boys and 41.4% for girls). It’s worth noting that boys appreciated much more than girls bread and pasta (80.0% vs. 55.5%) and fat meat (68.9% vs. 44.0%).

Regarding the socio-cultural attributes (Table 3), some of the food categories were considered, more than others, to be natural and typical of the local (regional) area and in some case of the countryside. These foods includes bread and pasta, cold cuts, dairy products, eggs, lean meat, oils and condiments, sweet fruit, and vegetables. Other foods like juices and drinks, fried potatoes, and fat meat were, on the contrary, more considered as being new, fashionable, and typical of an urban environment. Some differences between sexes were noticed regarding how the socio-cultural characteristics of fattening foods are perceived (Table 3). Of particular relevance is that girls (when compared to boys) consider some foods less fashionable (like pizza, pasta/bread, filled cakes/sweets, fried potatoes, biscuits and ice creams), and some foods more fashionable (in particular cold cuts and juices/drinks).

The present study assessed the beliefs of Italian children and adolescents about which foods are fattening, and evaluated the extent to which fattening foods are appreciated and how their socio-cultural attributes are perceived. Understanding how dietary habits develop in young people is important for planning effective nutritional intervention strategies. In fact, it is believed that nutritional habits tracks into adulthood from childhood (Nicklas et al. 1991, Boulton et al. 1995), and establishing healthy eating patterns at a young age can support nutritional habits in the following stages of the life (Kranz et al. 2004). Previous studies investigating this topic assessed the association between food marketing exposure and food choice, suggesting that exposure to television food advertisements and other marketing sources are linked to adolescent food choice and eating behaviours (Bannon and Schwartz 2006, Kelly et al. 2010, Scully et al. 2012). Furthermore, increased television watching has been associated to decreased nutritional knowledge and reasoning (Bannon and Schwartz 2006, Harrison and Marske 2005). An essential novelty of the present investigation is that it is specifically focused on the perception of fattening foods in a youth population. These results, on the whole, show a poorer dietary knowledge of children with respect to adolescents, and a different perception and appreciation of fattening foods in boys with respect to girls.

An association between age class and food category has been detected, suggesting that children and adolescents, when compared each to the others, indicate more or less frequently some specific foods or food categories. An examination of the percentage distributions of food categories (Table 2) reveals that children indicate, in some cases, foods that are not actually fattening, such as vegetables, non-sweet fruit and cereals, or lean meat. Conversely, children tend to rarely consider oils and condiments as fattening, whereas adolescents include more often these foods amongst fattening foods. These results clearly reveal a better dietary knowledge of adolescents, supporting that students have more opportunities of nutritional education as they advance in their scholastic experience, in agreement with what postulated by Yoon et al. (2008).

The present results show a clearly different perception of fattening foods in the two sexes. Overall, boys demonstrate a higher appreciation of fattening foods when compared to girls. This is in agreement with the inverse relationship between children’s perceptions of the healthiness of food and their preference of them, described by Noble et al. (2000). Specifically, boys appreciate more than girls the fat meat, fried potatoes, pizza, and especially bread and pasta. Furthermore, bread and pasta constitute 9.2% of the fattening foods indicated by girls, whereas boys consider such a category as fattening food only in 4.4% of cases. With high probability, girls are much influenced by popular dietary recommendations about the need to limit carbohydrate intake, which in the Italian diet are usually represented by bread and pasta. In fact, at these ages, girls take care to their figure and look much more than age-matched boys. This is demonstrated, for example, by Argnani et al. (2008), reporting that, in Italy, the ideal body image is larger in boys than in girls at the age of 8 and 9. Similarly to a previous study (Toselli et al. 2010), we observed a higher proportion of underweight subjects among girls (especially adolescents) than among boys, as well as a lower proportion of obese and overweight. Several authors (Marlett et al. 2002, Kaline et al. 2007, Mellen et al. 2008, Alexy et al. 2010) have reported the health effects of whole grains with respect to heart disease, cancer, gastrointestinal health, diabetes, and weight management studies. Despite in most cases these health effects have only been shown for adults, an elevated whole-grain intake may be desirable already in early life, as dietary habits develop during the early childhood and can track until adulthood (Lake et al. 2006). It was shown, using dietary surveys, that in most cases the intake of whole-grain is too low in adults (Cleveland et al. 2000, Thane et al. 2005) as well as in children and adolescents (Brady et al. 2000). Alexy et al. (2010) also reported a negative age trend of whole-grain intake. Therefore, it is important to prevent the risk that girls, aiming to reduce their weight, turn to self-made diets lacking in carbohydrates.

Further differences between sexes were observed regarding the perception of socio-cultural attributes of foods, and in particular of fashionability. Indeed, girls considered fattening foods (both overall and with reference to many specific food categories) as less fashionable when compared to their male counterparts. This may be due to higher exposure to advertisements showing fattening foods in a modern environment.

Finally, no association was revealed between the weight status and food perception. Therefore, despite the perception of foods affects the nutritional behavior and thus may contribute to deviations from the condition of normal weight, one individual’s weight status is more directly determined by other organic or psychological factors.

## Conclusions

In conclusion, this study provides important insights concerning the perception of fattening foods in the Northern Italian youth population. The present findings can serve for future research and potential nutritional interventions in children and adolescents. Further research should be carried out in other populations to identify how food perception is affected by cultural and social factors.

## Methods

The sample consists of 549 subjects, attending the primary school (children, aged 8–11; F: 125, M: 119) and the middle school (adolescents, aged 11–14; F: 153; M: 152) in Bologna, Emilia-Romagna, Northern Italy. The participants were randomly selected, and interviewed in the school year 2008–2009. The school institutions involved provided their approval to conduct the study, and parental informed consent was obtained for each participant.

Body height and weight were measured and the BMI (weight in kilograms divided by height squared in centimetres) was computed. According to his/her BMI, each subject was classified as underweight, normal weight, overweight, or obese, using International Obesity Taskforce (2012) thresholds for sex and exact age. Underweight was determined using the thresholds of Cole et al. (2007).

An individual oral interview was conducted, in which each participant was asked to indicate through an open choice five foods having, according to the individual opinion, a fattening effect. Then, for each of the five indicated foods, a yes-no answer was required to the question “is this food special for you?”, concerning the subject’s appreciation of the food, and to six questions of the type “is this food ….?”, concerning socio-cultural attributes of the food: new, fashionable, natural, local, typical of the city, typical of the countryside. The questions have been adapted from a questionnaire developed by Ulijaszek in a pilot study regarding the environmental factors contributing to obesity (2007).

All the foods indicated, according to similarities in their composition, nutritional characteristics, and pattern of consumption by children and adolescents, were grouped in 18 categories. Each category was named as the most representative food(s) of that category: biscuits and ice creams; bread and pasta; chocolate and snacks; cold cuts; dairy products; eggs; fat meat; filled cakes and sweets; fish; fried fish; fried potatoes; juices and drinks; lean meat; non-sweet fruit and cereals; oils and condiments; pizza; sweet fruit; vegetables.

Data are reported as percentage values. Pearson’s Chi-square tests were used to assess the association between the examined categorical variables, namely age class, weight status, food category, appreciation and socio-cultural attributes of the food. The statistical significance was set at p < 0.05. The analyses were carried out separately for boys and girls. All the analyses were carried out using the R statistical software (R Development Core Team 2011).
